# Efficient Reduction of Food Related Mould Spores on Surfaces by Hydrogen Peroxide Mist

**DOI:** 10.3390/foods10010055

**Published:** 2020-12-28

**Authors:** Cathrine Finne Kure, Solveig Langsrud, Trond Møretrø

**Affiliations:** Nofima-Norwegian Institute of Food, Fisheries and Aquaculture Research, P.O. Box 210, N-1431 Aas, Norway; solveig.langsrud@nofima.no (S.L.); Trond.moretro@nofima.no (T.M.)

**Keywords:** moulds, disinfection, hydrogen peroxide

## Abstract

The aim of the study was to evaluate the fungicidal effect of a H_2_O_2_ mist generating system for disinfection of spores of six food-related moulds (*Alternaria alternata*, *Aspergillus flavus*, *Geotrichum candidum*, *Mucor plumbeus*, *Paecilomyces variotii*, and *Penicillium solitum*) dried on stainless steel. Exposure to H_2_O_2_ mist for 2 or 4 h lead to >3 log reduction in mould spores in the majority of the tests. The presence of the soils 2% skim milk or 3% BSA did not significantly alter the fungicidal effect, while the presence of raw meat juice had an adverse fungicidal effect against *Penicillium* and *Mucor* in two out of three tests. Fungicidal suspension tests with liquid H_2_O_2_ confirmed the effectiveness of H_2_O_2_ on reducing the mould spores. Both the surface test and the suspension test indicated that *P. variotii* is more resistant to H_2_O_2_ compared to the other moulds tested. The study shows the efficiency of H_2_O_2_ mist on reducing food-related mould spores on surfaces.

## 1. Introduction

Fungi are a major cause of spoilage of food since they have a great versality for growing substrates and conditions where other microorganisms are not able to grow [1]. Fungal spoilage cause quality reduction due to visible or invisible defects such as patches and spots, texture changes, off-odour, and off-flavour. Some of the fungi growing on different foods such as cheese, dry-cured meat and fruit juices, may also produce mycotoxins, which lead to a food safety issue.

Mould spoilage will depend on several factors, such as the type and number of mould cells, the properties of the food (nutrients, water activity, and pH), and how the food is stored (temperature and packaging). Different mould genera cause food spoilage depending on the type of food and how the foodstuff is produced. *Aspergillus*, *Penicillium*, and *Fusarium* are the three most frequently genera in spoilage of foodstuff in general [1]. However, a range of other fungi has also been associated with spoilage of food. *Alternaria* is one of the main toxigenic fungal genera found in cereals worldwide [2] and *P. variotii* is a heat-resistant mould and a common air-borne contaminant [3]. *G. candidum* has a worldwide distribution, is isolated from a variety of foodstuffs and is also known as “machinery mould,” due to its ability to colonize food-processing environments [3]. Different *Mucor* species are also common in food and indoor environment and have been observed as “cat hair” on soft cheese [4].

The air and surfaces in production plants often contain mould spores [5,6] and the air represents a major contamination source of products as cheese and dry-cured meat at different stages [7,8]. Airborne spores can contaminate the surface of products and equipment; thus, cleaning and disinfection of the production environment are crucial. Cleaning and disinfection of the production environment can reduce the level of mould spores on the food and hence reduce the spoilage of the product. Moulds are, in general, not particularly resistant to disinfectants used in the food industry, neither those based on tensides (e.g., quaternary ammonium compounds), oxidative compounds (e.g., peroxide, peracetic acid, and hypochlorite) or alcohols [9]. Which disinfectants that are most effective is not clear, as different studies report contradicting results. The choice of disinfection system will depend on more factors than the biocidal efficacy towards the problem organisms, such as robustness of the disinfectant to environmental factors (soil and temperatures) and an application method that reaches the organisms and ensure sufficient contact time.

Typical challenges with conventional foam/gel-based disinfection methods are that some areas may be difficult to reach (e.g., inside machines, ventilation systems), that some equipment may not withstand humid cleaning (e.g., electrical components) and that the effect is limited against air-borne microorganisms. A possible alternative or supplemental approach to conventional open foam-based disinfection is fogging disinfection, where the disinfectant is distributed in the processing environment as an aerosol or mist [10]. An example of fogging disinfection is the use of hydrogen peroxide (H_2_O_2_) mist. The concept of the method is that liquid H_2_O_2_ (usually with a concentration of 5–10%) is pumped through a nozzle, which produces small droplets that will evaporate to gas form and be spread to the surroundings. Several types of H_2_O_2_ mist-based commercial systems exist and the concept is currently used and has been frequently tested in health care environments against bacteria [11,12]. For use in the food industry, less information is available, but we have previously shown that H_2_O_2_ mist is effective against the foodborne bacterial pathogen *Listeria monocytogenes* [13]. There is limited information available about the effect against moulds, but H_2_O_2_ mist was found to be effective against moulds in indoor air in a dairy [14] and against *P. digitatum* on wood sticks in citrus storage rooms [15]. Another approach for fogging disinfection with H_2_O_2_ is the use of H_2_O_2_ vapour. This differs from the use of H_2_O_2_ mist, using a more concentrated H_2_O_2_ solution (30–35%), which is vaporized by the use of heat, usually 40 °C. Use of H_2_O_2_ vapour/mist has been shown to reduce moulds and decay on fresh produce and plants [16,17,18].

The aim of the study was to evaluate the fungicidal effect of a H_2_O_2_ mist generating system (Decon-X) for disinfection of food-related mould spores on surfaces. The disinfection effect was tested on both clean and soiled surfaces.

## 2. Materials and Methods

### 2.1. Mould Strains

The fungi used in the present study and their origin are shown in Table 1. Six different moulds representing different genera commonly isolated from different food products were studied. The strains used were isolated from different food and nonfood products and identified using traditional methods and ITS sequencing [3]. The strains were stored at −80 °C.

### 2.2. Preparation of Mould Spore Suspensions

Malt extract agar (MEA) (Oxoid, Hampshire, United Kingdom) plates were inoculated with fungi from freezing stocks. The agar plates were incubated 7–9 days at 25 °C, except for *M. plumbeus*, which were grown at 15 °C. Spore suspensions were made according to a protocol described previously [19], briefly as follows. Suspension of conidia (hereafter called spores) were made by adding 25 mL 0.05% Tween 80 (Sigma-Aldrich, Saint-Louis, MO, USA) to the culture plate, followed by scraping with a sterile L-shaped spreader (VWR International, Radnor, PA, USA. Then, each spore suspension was vortexed for 30 s in a 50 mL centrifuge tube (Sarstedt, Nümbrecht, Germany) with 8× *g* of sterilized glass beads (no. 1401/2; Assistant) and filtered through sterile glass wool (ACROS Organics, Geel, Belgium).

### 2.3. Exposure of Mould Spores to H_2_O_2_ Mist

For each of the six mould strains (Table 1), four drops of spore suspension (10 µL each) were applied to a stainless-steel coupon of 20 mm × 20 mm (AISI 304, 2B, Norsk Stål AS, Nesbru, Norway), placed in a petri dish without lid, and incubated for 30 min to 1 h, until visibly dry, in a safety hood. The number of spores added on each coupon varied in the range of 3.5–6.2 log. Two parallel steel coupons were inoculated with mould spore suspension for each test. The petri dishes with the mould-containing coupons were placed on a conveyor belt, about 80 cm above the floor in a 36 m^3^ test room. The room was disinfected with H_2_O_2_ mist using of a Decon-X DX1 machine (Decon-X International, Lysaker, Norway). A 5% (50,000 ppm) H_2_O_2_ solution (Decon-X 520/521, Decon-X International) was used for mist generation. The disinfection process was performed as described previously, where H_2_O_2_ mist was automatically produced when the relative humidity was <90%, leading to a H_2_O_2_ concentration in the air in the range of 40–80 ppm during the disinfection process [13]. The H_2_O_2_ concentration in the air was measured with two sensors, i.e., one sensor on the outside of the mist generator and one sensor close to the samples, as described previously [13]. The temperature during the disinfection tests was 20–23 °C. The spores were exposed for 2 or 4 h. At the end of exposure, the lids were put on the petri dishes before they were removed from the test room by a person wearing a protective gas mask. Control coupons prepared the same way as for the coupons exposed to H_2_O_2_ were kept outside the disinfection room during the disinfection test.

To determine the number of surviving spores, the steel coupons were swabbed using cotton tipped applicators, single tip (Selefa, OneMed Group Oy, Dandervd, Sweden). One swab was moistened in Dey-Engley neutralization broth (Remel, Lenexa, MO, USA) before swabbing the entire surface of the steel coupon. The swab was put into a 14 mL Falcon round-bottom tube (Corning Science, Corning, New York, NY, USA) containing 2 mL Dey-Engley neutralization broth. The tube was vortexed, and the swab was discarded. The sample and serial 10-fold dilutions prepared in peptone water were plated on MEA incubated at 25 °C for 5–7 days and then counted, with the exception of plates with *M. plumbeus*, which were incubated at 15 °C. The tests were repeated three times at different days.

In all test runs with exposure to H_2_O_2_ mist, a commercial biological indicator with spores of *Geobacillus stearothermophilus* (Apex biological indicator 4–5–6 log, Mesa labs, Bozeman, MT, USA) was included for process control and placed next to the petri dishes with the steel coupons with the spores. The commercial spore test was analysed according to the manufacturers’ instructions.

### 2.4. Effect of Food Soils on the H_2_O_2_ Mist Disinfection Effect

To test the robustness of H_2_O_2_ disinfection of mould spores, spore suspensions of *M. plumbeus* and *P. solitum* were made in three types of food soils: 3% bovine serum albumin (BSA) (Sigma-Aldrich, Saint-Louis, MO, USA), 2% reconstituted skim milk (Merck, Kenilworth, IL, USA), and raw meat juice. The *M. plumbeus* strain was used since this strain was isolated from cheese (dairy product), and *P. solitum* was used since the strain was isolated from a meat product. BSA and skim milk are recommended as model soils to be used in standard disinfection tests [20,21]. Raw meat juice (prepared as described previously [13]) was included since it was previously found to quench the effect of H_2_O_2_ mist against *L. monocytogenes* [13]. As we did not want to mix food soils and 0.05% Tween 80 (as was used to prepare spore suspensions in the initial tests), spore suspensions were made by collecting the spores from plates with washing with food soils or dH_2_O (control). The spore suspensions were dried on stainless steel coupons and the coupons with the spores were exposed to H_2_O_2_ mist for 2 h and sampled as described in Section 2.3.

### 2.5. Fungicidal Suspension Test with Liquid H_2_O_2_

Fungicidal suspension tests with liquid H_2_O_2_ were performed according to EN 1650:1997 [20], with some adjustments. The spore suspensions of the six strains were made as described in Section 2.2. To evaluate if the age of the spore suspension could influence the sensitivity of the moulds, in some cases (in addition to fresh suspensions), also spore suspensions that had been stored at 4 or 20 °C for 14 days prior to the fungicidal test were tested. A volume of 0.5 mL spore suspension (corresponds to 6–8 log of spores) was added to 4.5 mL of dH_2_O (control) or liquid H_2_O_2_ (Sigma Aldrich, Saint-Louis, MO, USA), with a final H_2_O_2_ concentration of 4% or 6% (40,000 or 60,000 ppm). The concentrations were selected after initial tests showed the concentrations suitable for comparison of strains. After 15 min exposure at room temperature, 0.5 mL was transferred to 4.5 mL Dey-Engley Neutralizing broth, followed by dilution and plating to MEA. The plates were counted after 5–7 days incubation at 25 °C, except for plates with *Mucor* that were incubated at 15 °C.

### 2.6. Calculations and Statistics

The fungicidal effect of H_2_O_2_ mist exposure was calculated as the difference between the log number of the surviving moulds after H_2_O_2_ exposure and the log number of spores on control coupons that were kept outside the disinfection room. Similarly, in the suspension tests, log reduction was calculated as the difference in mould counts between samples exposed to H_2_O_2_ and samples exposed to dH_2_O (control). For replicates in the suspension test with reduction above the detection limit, reduction values equal to the detection limit were used in the calculation of the mean. Minitab (Minitab^®^ 19.2 2019, Minitab Ltd., Coventry, UK) was used for statistical tests. The general linear model and Tukey’s comparisons of means were used to test the significance of the differences between mould strains or preparation of spore suspensions. All tests were based on at least three biological replicates performed on separate days and with new spore suspensions.

For two moulds (*A. alternata* and *M. plumbeus*), one out of three biocidal suspension tests resulted in a reduction above the detection limit. Then, one additional test was performed, and statistical significance was tested both including (setting the value equal to the detection limit) and excluding the value exceeding the detection limit. The most conservative result among these two approaches was used for reporting the result. For *P. solitum*, two out of three experiments with 6% peroxide resulted in a reduction higher than the detection limit. This strain was excluded from comparisons between strains at 6%.

## 3. Results and Discussion

### 3.1. Reduction in Mould Spores after Exposure to H_2_O_2_ Mist

During all test runs, a concentration of H_2_O_2_ in the range of 40–80 ppm (after the initial filling phase) was measured in the air in test room. In addition, all test runs resulted in 5 log reduction in the biological indicator (*Geobacillus* spores). Together these control parameters confirmed a successful disinfection process for all test runs.

The results show that the hydrogen peroxide mist in the majority of the tests reduced the level of the different species of moulds with >3 log (Table 2), which is regarded as an efficient disinfection [21]. No spores were detected (>3 log reduction) after 4 h of exposure to peroxide mist in 15 out of 21 (71%) tests. Treatment for 2 h resulted in more than 3 log reduction in 12 out of 21 (57%) tests.

For *A. flavus*, *M. plumbeus*, and *P. variotii*, a variation in the fungicidal effect was observed between different test/days. The H_2_O_2_ mist disinfection was effective against the other moulds and the biological indicator (*Geobacillus* spores) in all test runs, and the H_2_O_2_ concentration in the air was measured to be within the same range (40–80 ppm) for all test runs. It still cannot be ruled out that small variations in the disinfection process led to the variation in results. However, it is more likely that the variation in the inactivation was associated with variation in the sensitivity of the mould spores to H_2_O_2_ mist. In a review of fungal spores and food mycology [22], Dijksterhuis points out that spore populations are heterogenous and contain spores of different age, history, and, henceforth, composition. This results in a broadening of the distribution of stress resistance, and a number of subpopulations may occur. Subpopulations of different spores produced by one species or even one colony can occur [23], and subpopulations of different spores may exist that show resistance to one stressor. Other studies point out that the lack of reproducible results with conidia could be due to the presence of a thinner cellular membrane that makes *Penicillium*, *Aspergillus*, and *Mucor* strains more sensible to chemical stresses [24] or by their inability to encode transcription factors required for stress tolerance (e.g., heat shocks or hydrogen peroxide), such that observed in *Aspergillus oryzae* by Sakamoto et al. [25]. Because of these variations, which may be larger in situ than in controlled laboratory test, it is crucial to perform disinfection experiments in systems close to practice and do real biological replicates to be able to conclude about effects. Unfortunately, from the methodological description, many studies use technical replicates or no replicates [9,26,27,28]. This will more likely result in statistically significant effects due to reduced variation, but any conclusions about how the method will perform taking into account natural variation cannot be drawn and the results will have limited value.

There are some other studies where H_2_O_2_ mist/vapour has been tested against food/food industry-associated moulds. In a study by Masotti et al. [14], air disinfection was tested by hydrogen peroxide mist for 16–20 min, and reductions of 0.7 and 1.2 log of moulds were found in two processing rooms. Lower reductions were obtained than in the present study, but there were several methodological differences (exposure time, test on surfaces vs. air, mist generation system, and mould types) between the two studies that may explain this. Unfortunately, no information was provided about the H_2_O_2_ concentration in the air in the room. In the same study, the effect of ozonation against moulds was about one log higher than as for H_2_O_2_ mist. Smilanick et al. [15] found that exposure for 3 h to H_2_O_2_ mist (different solutions with 26–30% H_2_O_2_ used for mist generation, with two mist generating systems tested) lead to a reduction in germination in the range of 50–95% of *P. digitatum* conidia on wooden craft sticks within citrus degreening rooms. Fogging with H_2_O_2_ was among the most effective among several types of fogging disinfectants tested. In another study, the effect of whole room disinfection with various fogging disinfectants, including H_2_O_2_, was tested on moulds present on strawberries and on moulds in the air in the room where the strawberries were stored [29]. The effect of H_2_O_2_ varied between experiments but was in the range of 0.5–2 log (increasing reduction with increased concentration of H_2_O_2_ solution used for mist generation). The H_2_O_2_ concentration in air in the room was not measured. The effect of whole room disinfection with H_2_O_2_ on moulds on strawberries was in the same range as for fogging with ethanol, chlorine dioxide, citric acid, and sodium hypochlorite. There are also other studies confirming a reducing effect of H_2_O_2_ mist/vapour on moulds and decay of fruits and vegetables [16,17,18], but direct comparison with the present study is difficult, since there were differences in temperatures, concentrations, exposure time, etc. between the studies. When fruits/vegetables are exposed directly, it should be noted that miscolour after treatment may be an issue for some products, e.g., grapes [16]. Although direct comparison between the present and the other studies described above are difficult due to many methodological differences and the lacking of H_2_O_2_ concentration measurements in the other studies; together, the studies confirm that H_2_O_2_ mist/vapour has a potential for control of moulds in the food industry.

### 3.2. Influence of Food Soils on the Fungicidal Effect of H_2_O_2_ Mist Disinfection

Spores of *M. plumbeus* and *P. solitum* suspended in three types of food soils were dried on steel and exposed to H_2_O_2_ mist. The presence of 2% skim milk or 3% BSA did not significantly alter the disinfection effect against neither *Penicillium* nor *Mucor*. This shows that the H_2_O_2_ mist disinfection was robust in presence of proteins and fats, as described previously for *L. monocytogenes* [13]. The presence of raw meat juice had an adverse effect on the disinfection against *Penicillium* and *Mucor* in two out of three tests (for detailed results, see the Supplementary Material). In a previous study, it was shown that H_2_O_2_ was degraded in liquid meat juice, probably due to the presence of catalase [13], leading to a limited disinfection effect against *L. monocytogenes*.

### 3.3. Effect of Liquid H_2_O_2_ on Mould Spore Suspensions

**Figure 1:**
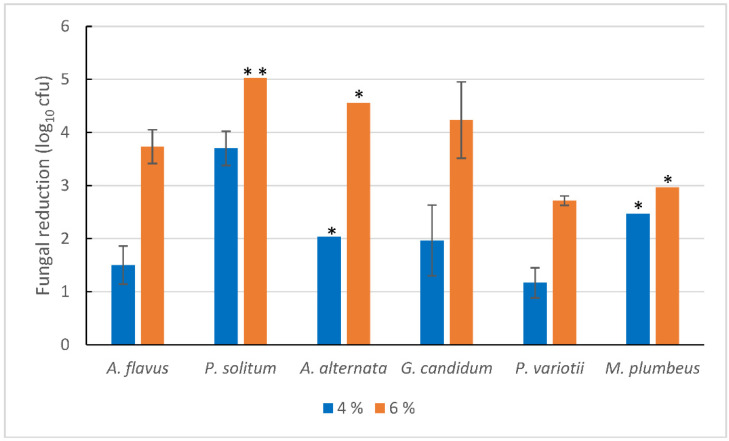
Reduction in log cfu after exposure to 4% or 6% liquid H_2_O_2_ in a fungicidal suspension test (15 min at room temperature) for fresh spore suspensions. Mean values and standard error of the mean of three to four experiments shown. Asterisks indicate number of replicates with mould counts below the detection limit (reductions above the detection limit). Standard error not shown for strains with number of surviving cells below detection limit in one or more of the replicates.

Results from the fungicidal suspension tests of fresh spores with liquid H_2_O_2_ are shown in Figure 1. Overall, the reductions were higher in 6% than 4% liquid H_2_O_2_ (*p* < 0.001). The inactivation differed between strains exposed to 4% hydrogen peroxide (*p* = 0.02), with *P. variotii* and *A. flavus* being significantly less susceptible than *P. solitum* (Tukey, *p* < 0.05). No other pairs of strains were significantly different. For exposure to 6% liquid hydrogen peroxide, no differences were found, and with the exception of *P. variotii* (mean log reduction in 1.9), the reduction was close to or higher than 3 log. An initial experiment with all strains to test whether storage of spores (14 day, 4 or 20 °C) affected the susceptibility to hydrogen peroxide mist showed no effect with the exception of *A. flavus*. For *A. flavus*, further replicates were performed and the reduction was higher for spores stored at 4 or 20 °C for 14 days, compared to fresh spores exposed to 4% H_2_O_2_ (approximately, 2.5 log difference for both temperatures, *p* = 0.008) and 6% H_2_O_2_ (approximately, 1 log difference in reduction, *p* = 0.13).

Scaramuzza et al. [24] reported partially or totally inactivation of fungal conidia of *P. solitum*, *Aspergillus brasiliensis*, and *Mucor circinelloides* on laminated surfaces or in suspensions after exposure for up to 30 s for 40% liquid hydrogen peroxide at 25 °C, but the authors stated that the reproducibility was low. However, ascospore-forming strains (*Talaromyces bacillisporus, Aspergillus hiratsukae*, and *Chaetomium globosum*) were not affected by hydrogen peroxide, even when increasing the exposure time to 10 min. Bundgaard-Nielsen et al. [9] found poor effect by exposure to 3% hydrogen peroxide for 10 min in suspension tests against fungal contaminants commonly found in bread and cheese manufacturing. However, compared to our study, the concentration used was lower and the exposure time was shorter.

Both the surface test and the suspension test indicate that *P. variotii* is more resistant to the hydrogen peroxide compared to the other mould species tested. This species is able to produce airborne conidia that is more heat resistant than similar species [23] and are well known because of high heat and chemical resistance [30].

## 4. Conclusions

The results show that H_2_O_2_ mist is efficient for reduction in mould spores belonging to several genera that are common contaminants in food and food-processing environments. The reduction was >3 log of mould spores on stainless steel after 2 or 4 h in the majority of the tests. The reason for the observed variation in disinfection effect is not clear but may be due to the variation in the sensitivity of the spores. The results show that the effect of the H_2_O_2_ mist is robust in presence of proteins and fat but reduced in presence of raw meat.

Hydrogen peroxide was effective against food-associated mould spores on stainless steel in laboratory tests and may be suited to disinfect areas that may be difficult to reach (e.g., inside machines and ventilation systems) and equipment that do not withstand humid cleaning (e.g., electrical components) in the food industry. However, to further validate this potential, future studies should include testing against food-associated moulds in indoor air and practical tests in the food industry.

## Figures and Tables

**Table 1:** Mould strains used in this study.

Mould Species	Nofima Strain Collection Number	Origin
*Alternaria alternata*	MF07134	Wheat
*Aspergillus flavus*	MF04921	Food waste
*Geotrichum candidum*	MF04935	Horse skin
*Mucor plumbeus*	MF07127	Cheese
*Paecilomyces variotii*	MF04901	Food
*Penicillium solitum*	MF07110	Production environment, dry cured meat

**Table 2:** Fungicidal effect of H_2_O_2_ mist disinfection against mould spores dried on stainless steel.

Mould ^§^	Log Reduction					
	2 h *			4 h *		
	Replicate 1	Replicate 2	Replicate 3	Replicate 1	Replicate 2	Replicate 3
*Alternaria alternata*	>3.0 ^†,‡^	>3.0	>3.0	>3.0	>3.0	>3.0
*Geotrichum candidum*	2.1	2.8	>3.0	>3.0	>3.0	>3.0
*Paecilomyces variotii*	>3.0	2.4	−0.4	>3.0	>3.0	0.2
*Aspergillus flavus*	0.1	>3.0	>3.0	0.64	>3.0	>3.0
*Penicillium solitum*	>3.0	>3.0	>3.0	>3.0	>3.0	>3.0
*Mucor plumbeus*	2.8	>3.0	>3.0	>3.0	>3.0	>3.0

* Exposure time; ^†^ Numbers given are log_10_ reduction for three replicates performed at different days. Numbers in same columns are not always from experiments performed on the same day; ^‡^ Results from tests where the number of surviving spores were below the detection limit are presented as >3 log reduction; Disinfectants with >3 log reduction are recognized as effective against mould spores [21]. **^§^** Number of spores added on each coupon varied in the range of 3.5–6.2 log.

## Supplementary Materials

The following are available online at https://www.mdpi.com/2304-8158/10/1/55/s1, Table S1, Fungicidal effect of H_2_O_2_ mist disinfection (2 h) against mould spores in food soils or dH_2_O (control) dried on stainless steel.
