# Supplementary material for: Efficient Reduction of Food Related Mould Spores on Surfaces by Hydrogen Peroxide Mist
Source: Foods. 2020 Dec 28;10(1):55. doi: 10.3390/foods10010055 (PMC7823841; doi:10.3390/foods10010055)
Supplement: Supplementary file 1 [file foods-10-00055-s001.pdf]

Supplementary File

Table S1. Fungicidal effect of H_2O_2 mist disinfection (2 h) against mould spores in food soils or dH_2O (control) dried on stainless steel.

Mould/Soil	Log Reduction											
	qdH_2O (Control)			2% Skim Milk			3% BSA			Meat Juice		
	1 *	2	3	1	2	3	1	2	3	1	2	3
Penicillium solitum	>3.0	>3.0	>3.0	>3.0	>3.0	>3.0	>3.0	>3.0	>3.0	>3.0	0.63	0.26
Mucor plumbeus	1.66	1.79	>3.0	1.13	0.7	>3.0	2.72	1.8	>3.0	2.09	0.67	2.09

* Three replicates perform for each treatment.
